# CT and MRI Findings of Hepatic Involvement in Rendu-Osler-Weber Disease

**DOI:** 10.1155/2012/484085

**Published:** 2012-11-07

**Authors:** Mehmet Bilgin, Seyma Yildiz, Huseyin Toprak, Issam Cheikh Ahmad, Ercan Kocakoc

**Affiliations:** Department of Radiology, Faculty of Medicine, Bezmialem Vakif University, Adnan Menderes Bulvari, Vatan Caddesi, Fatih, 34093 Istanbul, Turkey

## Abstract

Rendu-Osler-Weber disease is a rare autosomal dominant disorder. Hepatic involvement manifests itself as vascular, parenchymal, and biliary lesions with characteristic telangiectasias and vascular shunts. In a 37-year-old female patient, dynamic contrast-enhanced upper abdominal CT and MRI were performed. CT and MRI revealed dilated celiac trunk and hepatic artery. On early arterial phase, dilated hepatic veins showed significant enhancement. On arterial and portal venous phases, liver showed significantly heterogeneous contrast enhancement and showed homogenous enhancement in the hepatic parenchymal phase. On the magnetic resonance cholangiopancreatography, irregular biliary ducts with strictures and dilatation were seen.

## 1. Introduction

Hereditary haemorrhagic telangiectasia (HHT), also known as Rendu-Osler-Weber disease, is a very rare hereditary autosomal dominant vascular disorder that occurs with an estimated frequency of 1–20 cases/100,000 [1]. Hepatic involvement occurs in up to 31% of cases and consists of vascular, parenchymal, and biliary lesions with characteristic telangiectasias and vascular shunts [2]. Patients with hepatic involvement can be asymptomatic, but congestive heart failure, portal hypertension, portosystemic encephalopathy, cholangitis, and atypical cirrhosis have been reported in the literature [2, 3]. Thus, diagnostic imaging is important in the identification of liver changes. The gold standard for diagnosing liver involvement in HHT is selective digital subtraction angiography (DSA) of the hepatic artery; however, it is an invasive method for the screening and diagnosis. Therefore, computerized tomography (CT) and magnetic resonance imaging (MRI) play a central role in the visualization of hepatic changes [4]. There were few reports about the value of MRI in this disease, despite the fact that this technique allows imaging of the liver parenchyma, biliary tract, and hepatic vessels. Magnetic resonance cholangiopancreatography (MRCP) is a noninvasive technique in the detection and characterization of bile duct abnormalities [2]. 

## 2. Case Report 

37-year-old woman with HHT who had been followed for 7 years were admitted to our hospital with jaundice and respiratory failure. Physical examination revealed cyanosis of the lips, hyperventilation, and expansion of the neck veins, icteric skin and sclera, anasarcalike edema of the body, and dermatitis in the legs due to edema. Laboratory findings revealed increased total bilirubin of 23 mg/dL and blood gas values: pO_2_: 48 mmHg, pCO_2_: 27 mmHg, SO_2_: 89%. Echocardiography demonstrated right atrium and ventricle dilatation. Right cardiac catheter angiography examination showed signs of severe pulmonary hypertension. The patient primarily received vasodilatory therapy (Sildenafil) and intensive diuretic therapy. Within 3 days, 10 kg reduction in her body weight and obvious regression of her symptoms were seen. She underwent contrast-enhanced chest CT, dynamic contrast-enhanced upper abdomen CT, and abdomen MRI/MRCP examination. Chest CT examination shows 5 opacities measured 5–15 mm in the left lung basal region; they were considered as arteriovenous shunts. Pulmonary trunk, pulmonary arteries, right atrium, and right ventricle appeared dilated. Contrast-enhanced dynamic CT images were acquired at 15 (early arterial phase), 30 (late arterial phase), and 120 seconds (late venous phase) after bolus injection of contrast medium. Contrast-enhanced dynamic MR-imaging was initiated at 25 seconds (arterial phase), 55 seconds (portal phase), and 150 seconds (late venous phase) after the start of the bolus injection of contrast medium.

**Figure 1:**
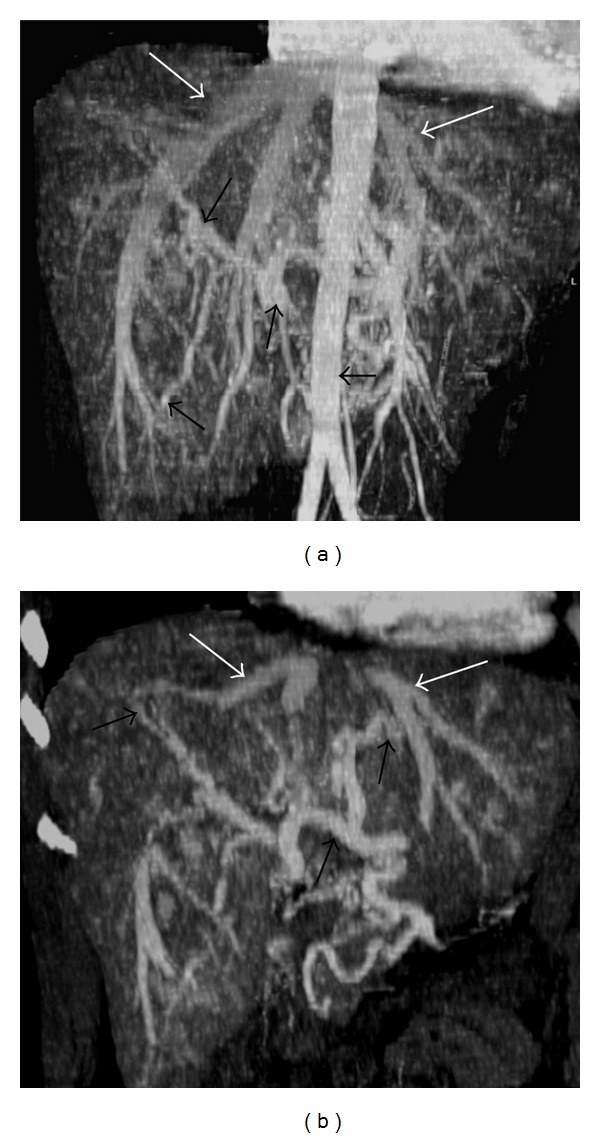
CT with coronal maximum intensity projection (MIP) reconstruction at early arterial phase (a, b) shows enlargement and early enhancement of the hepatic venous branch (white arrow), due to arteriovenous fistula and a large hepatic artery is also visible (black arrows). Abdominal aorta (arrowhead).

**Figure 2:**
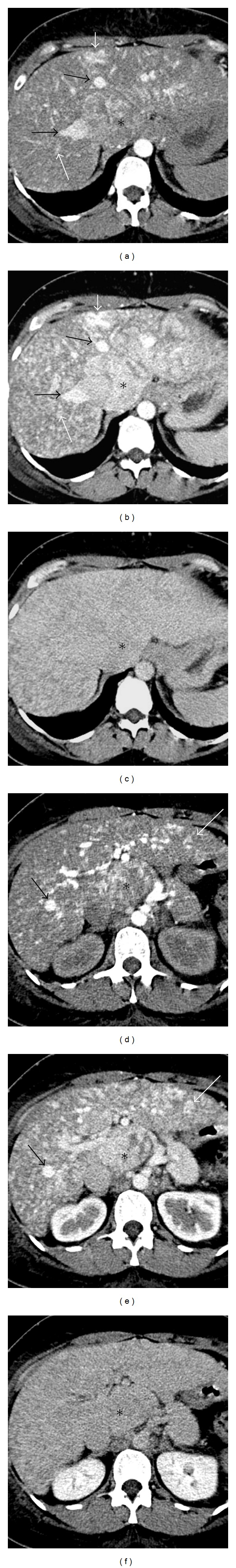
Axial CT scans obtained from different levels in the early arterial phase (a, d), late arterial phase (b, e) and late venous phase (c, f). During the arterial phases, millimetric hypervascular images disseminated throughout the hepatic parenchyma, referring to telangiectases that are no longer evident during the late venous phase are seen (white arrows). The early venous drainage with the simultaneous opacification of the dilated hepatic veins (black arrows) and the hepatic artery is evident during both arterial phases. Large confluent vascular masses are evident in the caudate lob (∗). A strongly enhancing subcapsular area with irregular shape, relating to an area of transient hepatic parenchymal enhancement (arrowheads), is evident in the fourth segment. These changes are no longer evident during the late venous phase.

On dynamic contrast-enhanced upper abdominal CT, dilatation of hepatic veins and significant contrast-enhancement during the early arterial phase was seen (Figures 1 and 2). The width of right hepatic vein was measured 27 mm. On arterial phase, multiple nodular hypervascular foci diffusely scattered throughout the liver consistent with arteriovenous shunts were seen. On early arterial and late arterial phases, significant heterogeneous contrast enhancement was seen due to disseminated intraparenchymal telangiectasias, hyperattenuating parenchymal areas and A-V shunts in the liver parenchyma. In caudate lobe, approximately 6 × 5 cm ball-shaped vascular formation which became more evident on late arterial phase was seen (Figures 2(a), 2(d), 2(b) and 2(e)). During the late venous phase, liver parenchyma showed homogenous contrast enhancement (Figures 2(c) and 2(f)). Celiac trunk and hepatic artery appeared dilated (the diameter of celiac trunk was 13 mm; the diameter of hepatic artery at hilus was 11 mm). Whereas the liver size was normal, the size of left lobe increased and the left lobe was hypertrophic compared to the right lobe and caudate lobe. Irregularity of liver contours and perihepatic mild amount of ascites were seen. 

**Figure 3:**
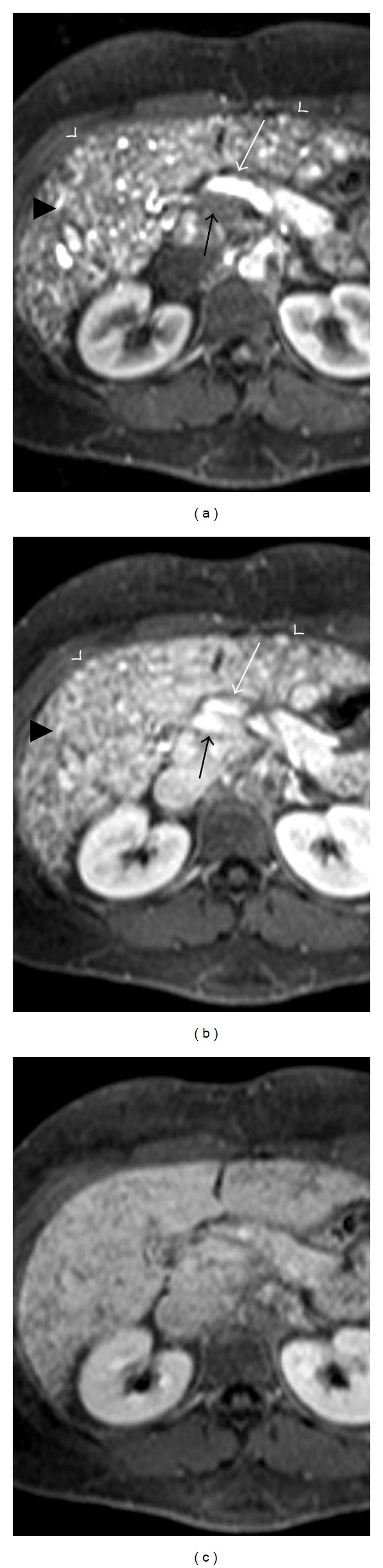
Axial dynamic MRI in the arterial phase (a), the portal venous (b), and the late venous phase (c). The early venous drainage with the simultaneous opacification of the right hepatic veins and the hepatic artery (black arrowheads) is evident during both arterial phases. The portal vein (arrow) is not opacified during arterial phase but it is opacified in portal phases. The hepatic parenchyma is inhomogeneous due to the presence of several millimetric hypervascular foci, consistent with telangiectasias (arrowheads). These inhomogeneities are no longer seen during the late venous phase.

**Figure 4:**
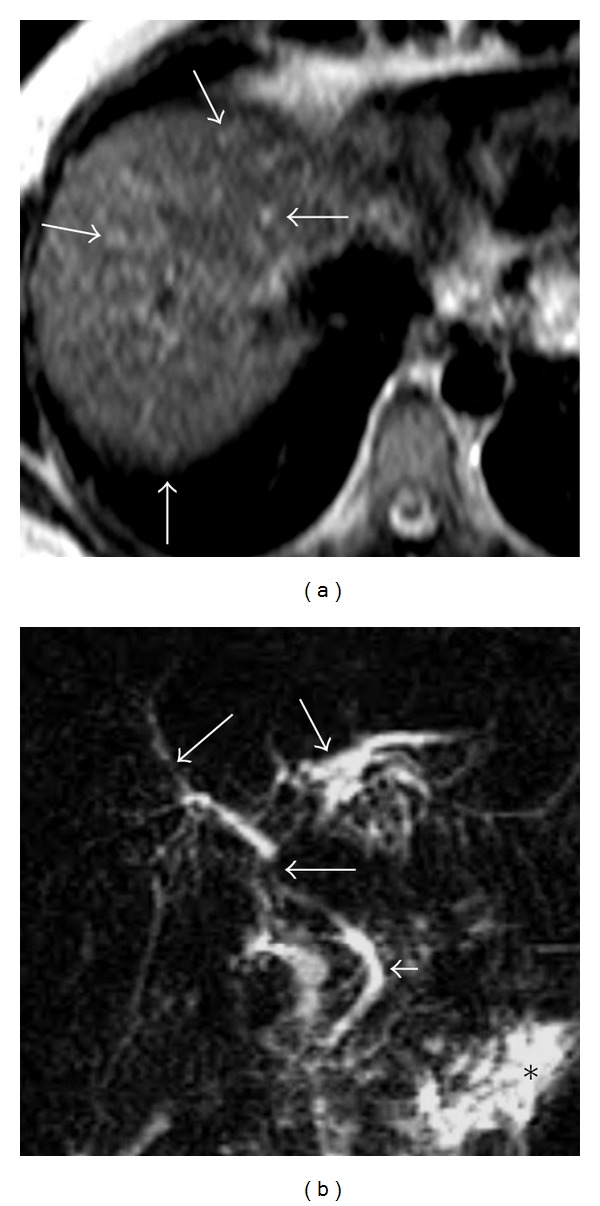
Axial T_2_-weighted sequence (a) shows multiple round lesions with high signal. The diameter of lesions is less than 10 mm, referring to telangiectasias (arrows). They are diffusely scattered throughout the liver and prevalently peripheral arrangement. MRCP sequence (b) shows contour irregularities multiple focal strictures and dilatation of the biliary tract (arrows) with a “pruned tree” appearance. Contour irregularity of the common bile duct wall is seen (arrowhead). (**∗**) Ascites.

MRI findings were similar to CT findings. The early venous drainage with the simultaneous opacification of the hepatic veins and the hepatic artery is evident during arterial phases. The portal vein was normal calibration and not opacified during arterial phase but it is opacified in portal venous phase. The hepatic parenchyma was inhomogeneous due to the presence of several millimetric hypervascular foci and A-V shunts (Figures 3(a) and 3(b)). These inhomogeneities are no longer seen during the late venous phase (Figure 3(c)). T_2_-weighted sequence shows multiple round lesions located peripherally with high signal. The diameter of lesions is less than 10 mm (Figure 4(a)). MRCP sequence showed contour irregularities, multiple focal strictures, and dilatation of the biliary tract with a “pruned tree” appearance (Figure 4(b)). 

## 3. Discussion

Liver involvement in HHT is frequent and characterized by the presence of intrahepatic shunts, disseminated intraparenchymal telangiectases, other vascular lesions, and bile duct abnormalities. US and color Doppler US are useful for detecting hepatic, and vascular lesions but should be completed by more accurate and sensitive imaging methods such as multidetector CT and MRI [5, 6].

On CT and MR imaging, arterial dilatation (a common hepatic artery greater than 7 mm in diameter) can be seen [7]. This increase in diameter is thought to be a consequence of an increased volume flow in the hepatic artery and veins caused by intrahepatic fistulas [2, 5, 7]. CT and MRI can show the prominent intrahepatic and extrahepatic arterial branches, dilatation of hepatic and portal veins and vascular shunts. Three types of intrahepatic shunts between the major vessels of the liver are possible: arteriosystemic (hepatic artery to hepatic vein), arterioportal (hepatic artery to portal vein), and portosystemic venous (portal vein to hepatic or systemic veins) [2, 3, 7]. Opacification of the hepatic veins during the arterial phase was considered as an indirect sign of the presence of hepatic-arteriosystemic venous shunt. Early enhancement of venous structures in the arterial phase suggests arteriovenous fistula. Arteriosystemic venous shunts are identified in 50 to 64% on conventional angiography in cases of liver HHT [2]. Early and prolonged enhancement of the portal vein during the arterial phase was considered an indirect sign of the presence of arterioportal shunt. Evidence of dilated portal veins communicating with the large systemic or hepatic vein during the portal-venous phase was considered a sign of intrahepatic-portosystemic venous shunt [2]. In our case, on early arterial phase, findings consistent with arteriovenous shunts presenting with intense enhancement of the dilated hepatic veins were observed. The diameter of the portal vein was normal, and there were no signs of portal hypertension.

On dynamic contrast-enhanced CT, on arterial phase, the early filling of hepatic and portal veins attracts attention. Also, on arterial phase, liver shows characteristically mosaic type heterogeneous perfusion pattern. The cause of this mosaic perfusion is multiple arteriovenous shunts showing different attenuation and telangiectasias. Telangiectasias are hypervascular rounded nodules with predominant peripheral location (varying from a few millimeters to 1 cm in size) in the arterial and late arterial phases, often becoming isointense in the hepatic parenchymal phase [7, 8]. These lesions appear high-signal intensity on T_2_-weighted MR sequences, and hypointense on T_1_-weighted sequences [2]. Telangiectases are seen in about 90% of HHT patients with liver involvement and are the elementary lesions of this disease [2, 3, 7]. 

Sometimes integrated confluent vascular masses appear as larger vascular pools (25%) and these masses characterized by early and persistent enhancement during the arterial phase [3, 7, 9]. Abnormal parenchymal perfusion was recognized in 65% of patients and referred to the transient hepatic parenchymal enhancement, reflecting the blood flow alteration induced by the shunt, and its evidence should be interpreted as another indirect sign of the presence of shunts. These hepatic perfusion disorders appear during the arterial phases as hyperattenuating parenchymal areas that become isoattenuating on venous phase images [7, 9]. 

HHT patients rarely present with cirrhosis secondary to extensive necrotizing cholangitis. The vascular supply of biliary ducts depends on the hepatic artery branches so shunts by stealing the arterial flow may cause ischemic cholangitis [10]. In the magnetic resonance cholangiopancreatography sequences, ischemic cholangitis was defined as irregular biliary ducts with strictures and upstream dilatation in the peripheral intrahepatic-biliary tract, with diffuse or segmental distribution or “pruned tree” appearance [2, 5]. On the other hand, bile duct obstruction and dilatation due to the compression of enlarged vascular structures have also been reported [2]. In our case, high bilirubin level with cirrhotic configuration of the liver suggests that cirrhosis secondary to necrotizing cholangitis, dilatation, contour irregularities, and multiple focal strictures in intrahepatic bile ducts were seen.

In conclusion, CT and MRI are the most accurate noninvasive modalities for the morphological study of lesions in HHT, and they have replaced angiography as diagnostic procedures. MRCP is a noninvasive technique in the detection and characterization of bile-duct abnormalities.
